# Mixed Reality Visualization of Radiation Dose for Health Professionals and Patients in Interventional Radiology

**DOI:** 10.1007/s10916-020-01700-9

**Published:** 2021-02-17

**Authors:** Takeshi Takata, Susumu Nakabayashi, Hiroshi Kondo, Masayoshi Yamamoto, Shigeru Furui, Kenshiro Shiraishi, Takenori Kobayashi, Hiroshi Oba, Takahide Okamoto, Jun’ichi Kotoku

**Affiliations:** 1 Graduate School of Medical Care and Technology, Teikyo University, Tokyo, Japan; 2 Diagnostic imaging Center, Kameda Medical Center, Chiba, Japan; 3 Department of Radiology, Teikyo University School of Medicine, Tokyo, Japan; 4 Central Radiology Division, Teikyo University Hospital, Tokyo, Japan

**Keywords:** Dose calculation, Interventional radiology, Mixed reality, Radiation dose, Visualization

## Abstract

For interventional radiology, dose management has persisted as a crucially important issue to reduce radiation exposure to patients and medical staff. This study designed a real-time dose visualization system for interventional radiology designed with mixed reality technology and Monte Carlo simulation. An earlier report described a Monte-Carlo-based estimation system, which simulates a patient’s skin dose and air dose distributions, adopted for our system. We also developed a system of acquiring fluoroscopic conditions to input them into the Monte Carlo system. Then we combined the Monte Carlo system with a wearable device for three-dimensional holographic visualization. The estimated doses were transferred sequentially to the device. The patient’s dose distribution was then projected on the patient body. The visualization system also has a mechanism to detect one’s position in a room to estimate the user’s exposure dose to detect and display the exposure level. Qualitative tests were conducted to evaluate the workload and usability of our mixed reality system. An end-to-end system test was performed using a human phantom. The acquisition system accurately recognized conditions that were necessary for real-time dose estimation. The dose hologram represents the patient dose. The user dose was changed correctly, depending on conditions and positions. The perceived overall workload score (33.50) was lower than the scores reported in the literature for medical tasks (50.60) for computer activities (54.00). Mixed reality dose visualization is expected to improve exposure dose management for patients and health professionals by exhibiting the invisible radiation exposure in real space.

**Supplementary Information:**

The online version of this article (10.1007/s10916-020-01700-9) contains supplementary material, which is available to authorized users.

## Introduction

In interventional radiology (IR), patient dose management generally uses only a displayed dose area product. However, this parameter does not identify the exposed position. For that reason, a dose distribution on the patient’s skin cannot be managed. Although, health professional exposure is administered using a personal dosimeter, the dose is unknown until the dosimeter is read out. To optimize exposure for all involved, health professionals must note their exposure dose in real-time according to each situation.

Several real-time dose estimation systems for a patient’s skin have been available [1–3]. However, the estimated dose distributions are displayed with a single value or as two-dimensional (2D) images. An intuitive real-time dose management system is expected to facilitate management of the dose and to improve patients’ and health professionals’ medical safety.

Monte Carlo (MC) method has been well known for its requirement of long durations to produce highly precise estimates. Recently, the MC method, which can be executed on a graphics processing unit (GPU) card for performing fast estimation, is reportedly used for dose calculation algorithms in IR [2, 4–6]. The method can calculate a patient’s skin dose distribution in real-time and can calculate the air dose distribution in a room rapidly and three-dimensionally (3D). Nevertheless, the calculated 3D information is typically degraded to 2D information simply to display it.

Recently, xR technologies such as virtual reality (VR) and mixed reality (MR,) are used in the medical field for applications related to surgical processes and education [6–9]. Earlier reports have described xR dose visualization systems for radiation dose notification [5, 6]. On these systems, a user can watch doses in virtual space. For that reason, it is unsuitable for clinical use during procedures. To produce new environments and visualizations in which physical and digital objects co-exist and interact in real time, MR technology provides merging of real and virtual worlds.

This study examined the concept and feasibility of a real-time dose visualization system using MR for dose management in IR: a Mixed Reality Dose Visualization System (MR-DVS). This report is the first of the relevant literature to propose an immersive MR radiation visualization system for IR.

## Materials and methods

### Design of a mixed reality visualization system

Our MR-DVS uses a wearable MR holographic device (Microsoft HoloLens; Microsoft Corp.). The HoloLens is a stand-alone MR hologram viewing platform. It uses a simultaneous localization and mapping (SLAM) algorithm for spatial mapping and detection of one’s own position. It provides an accurate 3D holographic representation of 3D digital objects with a full sense of depth. The objects can be viewed by a group of viewers simultaneously from all angles, enabling shared interaction. The display can be flipped up if it gets in the way.

Projection of the patient’s skin dose onto the patient’s body and estimation of the health professional’s dose requires accurate tracking of their positions and movements in a room. Nonetheless, position tracking using an external tracking system is adversely affected by disturbances such as the movement of staff members and fluoroscopy systems during a procedure. Therefore, an object recognition tracking method is preferred for MR-DVS. We designed MR-DVS on a 3D graphics platform (Unity 2017.4.3f1; Unity Technologies). For our application, we used object detection and tracking functionalities offered with a software development kit (SDK Vuforia; PTC Inc.) [10]. These technologies enabled us to register images in real time by tracking the relative position and orientation of the real objects using an RGB camera on HoloLens; this positional information is then used to update the transformations within the virtual world for dose projection and estimation. The optical detection and tracking of a target are useful for real-time high-accuracy registration with no need for an external tracking system [11, 12].

We used an image marker on Vuforia Engine as a tracking target. The engine detects and tracks the image features, which are stored in a preprocessed database. Once the tracking target is detected, the Vuforia Engine will track it so long as it is partially visible by the camera [10].

### Workflow

**Fig. 1:**
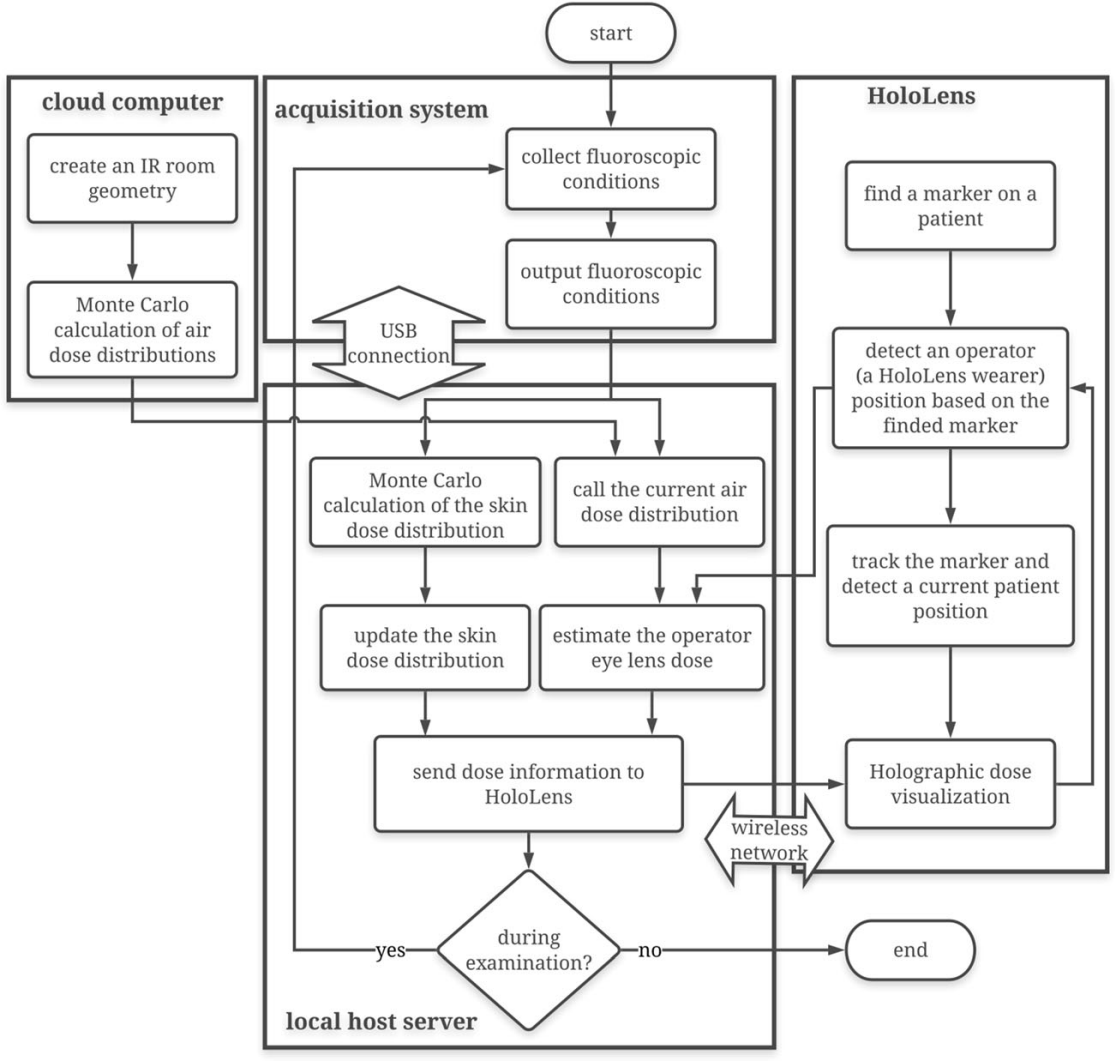
Flowchart of MR-DVS dose visualization using three separate computers: cloud computer for air dose calculation, localhost server for skin dose calculation and main data processing, and HoloLens for dose visualization and localization and mapping

Although HoloLens is a stand-alone device, it lacks sufficient computational power to run the real-time dose simulation. Therefore, we separated computation of the visualization and dose calculation respectively to HoloLens and an external server. The patient skin dose distribution and operator eye lens dose were estimated using the reported Monte Carlo system, designated as the fast dose estimation system for IR (FDEIR) [2] as described in the *Dose estimation system* section herein, on an external computer in real time. The estimated doses were transported sequentially to HoloLens and were projected in a real space. A flowchart of MR-DVS is shown in Fig. 1. It is described as the following.

**Fig. 2:**
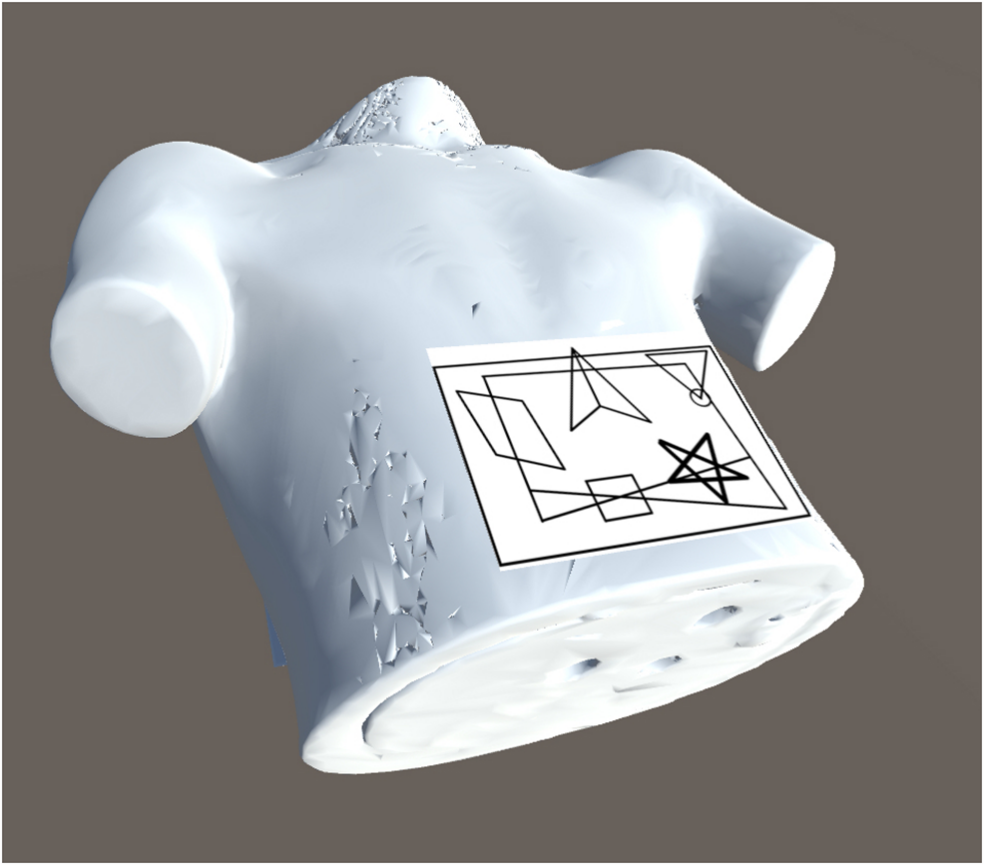
3D rendered chest CT images and the virtual recognition marker in Unity

Step 1: Users create an IR room geometry simulating their institution to calculate the air dose distributions in an IR room to reflect the behavior of scattered photons in the room. Even if a GPU card is used for dose calculation, air dose estimation requires long durations to produce highly precise estimates [5]. Therefore, we chose precalculation of air dose distribution to reduce the computational load for real-time dose estimation. For this study, we used a supercomputing system (Reedbush-H; Silicon Graphics International Corp.), at the Information Technology Center, The University of Tokyo, to calculate them efficiently. Precalculated dose distributions were stored in a dose table on the computer.
Step 2: Users virtually attach a recognition marker to a patient’s computed tomography (CT) images on Unity (Fig. 2). Then users attach the marker to the real patient body and recognize it using HoloLens. At this moment, the patient and user locations are detected and mapped to the virtual IR room for air dose estimation. Position tracking starts.
Step 3: Fluoroscopic conditions are collected from fluoroscopy systems, as described in the *Real-time acquisition of fluoroscopic conditions* section herein. Then the conditions are input into FDEIR and are used for eye lens dose estimation in real time. FDEIR calculates the patient skin dose distribution in real-time on a geometry generated from the patient’s CT images under current fluoroscopic conditions [2].
Step 4: MR-DVS detects a patient position through the recognition marker and a HoloLens position surrounding the user’s eye lens in 3D through SLAM and the marker. The recognized HoloLens position is transferred to the host server. Then, the eye lens dose at the present position is inferred from the precalculated table of air dose distribution under current fluoroscopic conditions, which was calculated in Step 1.
Step 5: The calculated skin dose distribution and the user’s eye lens dose are transferred sequentially to HoloLens. The skin dose is projected tightly on the patient body. The eye lens dose is displayed on a holographic screen.

### Dose estimation system

We used an earlier reported dose estimation system FDEIR for this study. It calculates a patient skin dose distribution and air dose distribution using the Monte Carlo method on a GPU card [2]. Compared to conventional-dose calculation systems, FDEIR more accurately estimates the skin dose distribution fitted to individual patients using the patient’s own CT images. Fluoroscopic conditions (field of view, source-to-image receptor distance, C-arm rotation, frame rate, kV, mA) are necessary for the calculation [2]. This system performs one skin dose simulation in a few seconds, which is much faster than conventional Monte Carlo calculations within 3.5% statistical uncertainty [2].

Moreover, FDEIRs can estimate the air dose distribution in a room. Estimated air doses have been consistent with measurements taken using dosimeters [4, 5]. The accuracy of FDEIR for skin and air dose estimation was validated in earlier studies [2, 4, 5]. Simulation geometries were constructed according to earlier studies [2, 5].

For real-time skin dose estimation, fluoroscopic conditions obtained using the acquisition system were inputted continuously to FDEIR during x-ray irradiation. The simulation was processed on a GPU card (Tesla K20c; NVIDIA Corp.) with a simulation of 10 million incident photons and 5 keV photon cut-off energy, as described in an earlier report [2]. The simulated dose distribution was projected sequentially on a patient body as colored holograms. For this study, we used CT images of a body phantom (PBU-10; Kyoto Kagaku Co., Ltd.).

However, FDEIR needs roughly one hour for air-absorbed dose calculation under a single condition [5]. Therefore, we precalculated and stored the air dose distributions in the IR room earlier under various fluoroscopic conditions on the Reedbush-H remote supercomputing system for MR-DVS. As in our example, doses were calculated under fluoroscopic conditions presented in Table 1 at each irradiation angle (−90° to 90° in lateral angle and − 30° to 30° between the cranial and caudal angles). Simulations were performed using FDEIR with a simulation of 10 billion incident photons and 5 keV photon cut-off energy, as described in an earlier report [5]. From the detected current user position and precomputed air dose distributions, health professional’s eye lens dose was conversion factors 1.550 Gy/Gy in International Commission on Radiological Protection Publication [5, 13].

**Table 1:** User rating for MR experience. A five-point scale (from 1 = strongly agree to 5 = strongly disagree) was used

	Mean rating (SD)		Median rating
It is easy to understand the patient skin dose distribution.	1.55	(0.94)	2
It is easy to understand your own exposure dose.	1.55	(1.00)	1
It is easy to perceive spatial relations between real and virtual objects.	1.95	(0.89)	2
You notice latency (lag, delay) between your own manipulation and virtual content.	2.85	(1.31)	2
The field of view is adequate for the application.	3.25	(1.21)	3
You experience postural discomfort during the application.	2.85	(1.42)	2
Hand manipulation is easy and intuitive.	1.75	(1.02)	2
Voice interaction is easy and intuitive.	1.95	(0.94)	2

### Real-time acquisition of fluoroscopic conditions

Calculation of doses in real-time on the continually changing fluoroscopic conditions requires importation of these conditions into FDEIR in real time. However, general fluoroscopy systems cannot transfer them outside the system in real time. Therefore, we built an acquisition system of conditions consisting of a number recognition system to recognize the displayed conditions on screens and an ultrasonic sensor system to detect the table positions for real-time dose calculation.

**Fig. 3:**
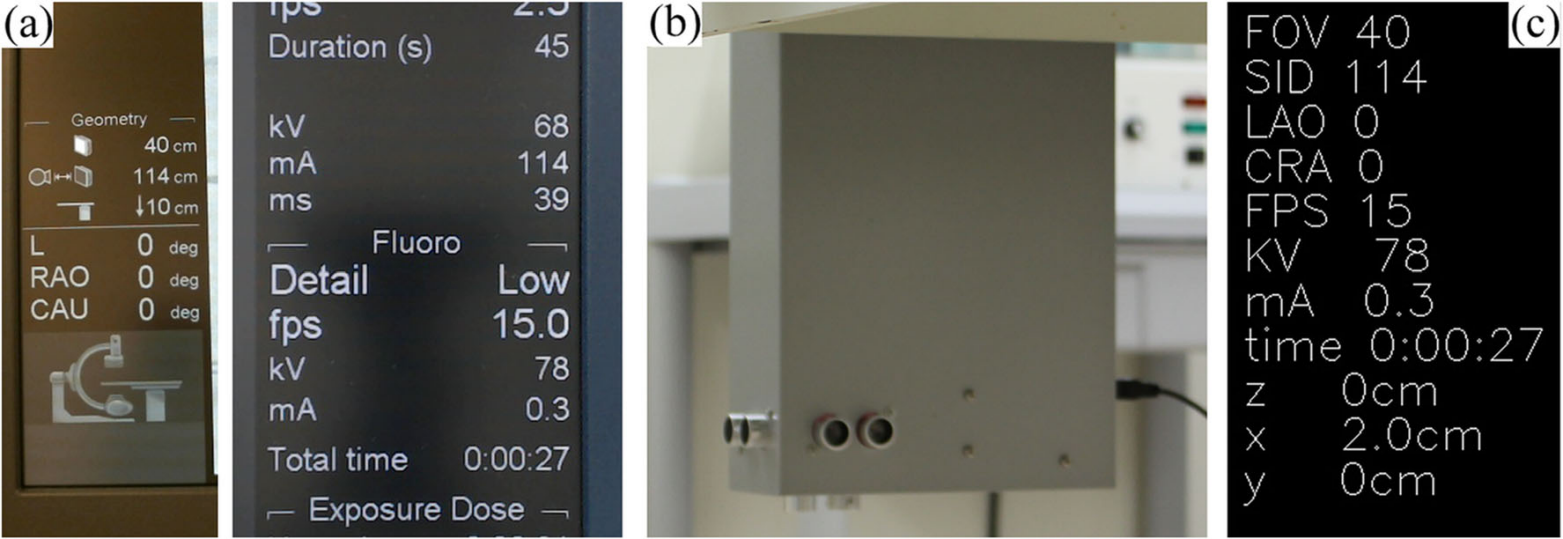
**a** Captured screen images from Innova 4100-IQ using web cameras for obtaining fluoroscopic conditions. **b** The ultrasonic sensor unit comprises three ultrasonic sensors facing three directions for measuring the table position. This sensor unit was installed under the table and was connected directly to the host server. **c** Recognized fluoroscopic conditions necessary for FDEIR from captured images and the sensor unit

In this study, a fluoroscopy system (Innova 4100-IQ; GE Healthcare) displays almost necessary fluoroscopic conditions for FDEIR on screens: Field of view, source-to-image receptor distance, C-arm rotation, frame rate, kV, mA, on/off of x-ray irradiation, and irradiation time. To obtain these fluoroscopic conditions from the displays in real time, we built a number recognition system using template matching (Fig. 3). Our number recognition system consists of web cameras placed in front of the displays, with the OpenCV library to achieve real-time image processing [14–17].

The table position was also a crucially important parameter to build an accurate simulation geometry. However, the fluoroscopy system (Innova 4100-IQ) reports the table position in the vertical direction only, with neither longitudinal nor lateral direction. Therefore, we also built a table position detection system using ultrasonic sensors to obtain a position in three directions (Fig. 3). This system comprises a microcontroller (Arduino Uno; Arduino, LLC) to control sensors, a temperature sensor (DHT11; D-Robotics) to correct sound velocity, and three ultrasonic sensors (HC-SR04; Elecfreaks Inc.) facing in three directions.

To validate the distance measurement accuracy, we compared measured values of ultrasonic sensors with that of a high-accuracy (100 μm) laser displacement sensor (IL-2000; Keyence Co.). Verification of the accuracy was conducted in each direction. The measurement was done at 2 cm intervals in the range of ±10 cm from a reference point.

The number recognition system and the table position detection system were synchronized. Then they were connected directly to the host server. Fluoroscopic conditions outputted from them were converted to an input file for the dose estimation system using an in-house Python program.

### Qualitative assessment

Qualitative tests were conducted to elucidate the workload, usability, and dose understandability of MR-DVS. This study examined 20 participants, comprising 2 radiologists and 18 radiological technologists, to assess MR-DVS. Before the assessment, participants learned how to interact with HoloLens using display-eye calibration and tutorial applications provided by Microsoft Corp. For the assessment, a virtual x-ray beam was exposed. Participants watched the virtual skin dose distribution and virtual exposure dose, which are the same as when the radiation was exposed.

The NASA Task Load Index (NASA-TLX) questionnaire and the Likert scale were used to evaluate the MR experience. As a subjective workload assessment tool, NASA-TLX was used to identify the primary score of the workload during the MR-DVS execution. NASA-TLX allows users to perform subjective workload assessments of health professionals. NASA-TLX derives an overall workload score between 0 and 100 based on a weighted average of ratings on six subscales: mental demand, physical demand, temporal demand, performance, effort, frustration level [18].

The questionnaire, which is presented in Table 2, was used to evaluate the system using a five-point Likert scale.

**Table 2:** Fluoroscopic conditions of the X-ray beam used for this study (Innova 4100-IQ)

Parameter	
kV	76 kV
mA	2.5 mA
Field of view	20 × 20 cm^2^
Frames per second	15
Source to image-receptor distance	100 cm
Source to surface distance	60 cm (at PA)
Filter	0.3 mmCu

## Results

**Fig. 4:**
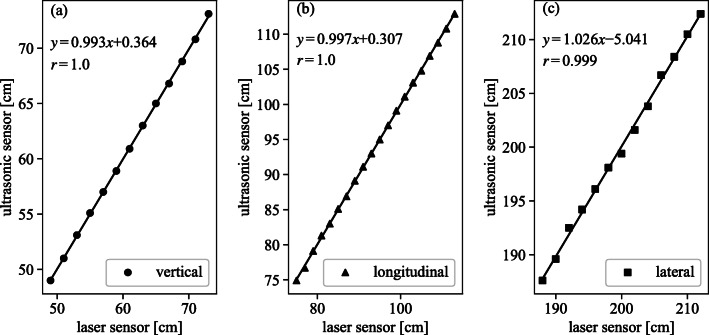
Comparison of the measured distance between the ultrasonic sensor and laser sonsor in each direction. Measured values were normalized at the reference points

The number recognition system accurately recognized all displayed fluoroscopic conditions every 0.2 s. Validation of the measured values of the sensor unit was done by comparison with the laser sensor value. The measured values, normalized at the reference points, are presented in Fig. 4. Averages of the error were less than 0.18% in three directions between our sensors and the laser displacement sensor.

**Fig. 5:**
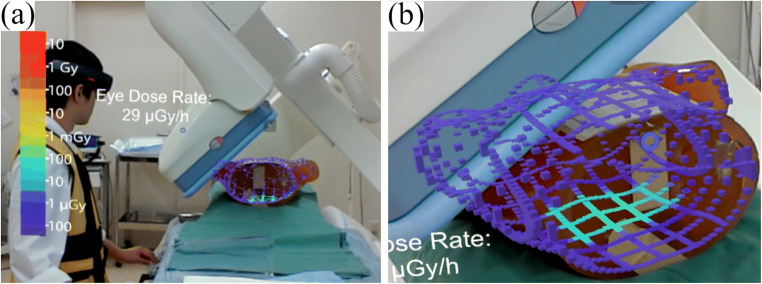
Mixed reality holograms of MD-DVS. (**a**) The left image shows the HoloLens user view, with appearance sharing and similar looking skin dose holograms with another HoloLens user. The value shown on the center represents the user’s own eye lens exposure dose at the current position. The color bar shows the patient skin dose levels. (**b**) The right image shows projected skin dose distribution. The high-dose area on the back in 3D did not obscure the background

An MR-DVS overview is presented in Fig. 5 and Video 1. One patient skin dose distribution was estimated within 3 s on average. The dose distribution was visualized with 4 s latency. It is depicted as colored cubes on the skin. A color bar shown in the air represents the absorbed dose level. The health professional’s eye lens dose rate was updated continuously. It was indicated as numerical values in the air. MR-DVS has the following functions.

- Tracking the patient body and keeping dose holograms on the body.
- Moving dose holograms to any space in the air and putting it back on the body.
- The x-ray frustum is shown as acquired from fluoroscopic conditions.
- Sharing holograms, HoloLens users can see the same holograms.
- MR-DVS kept showing the eye lens dose rate under the latest fluoroscopic conditions to notify the user of the exposure risk at a current position before the actual exposure.

**Fig. 6:**
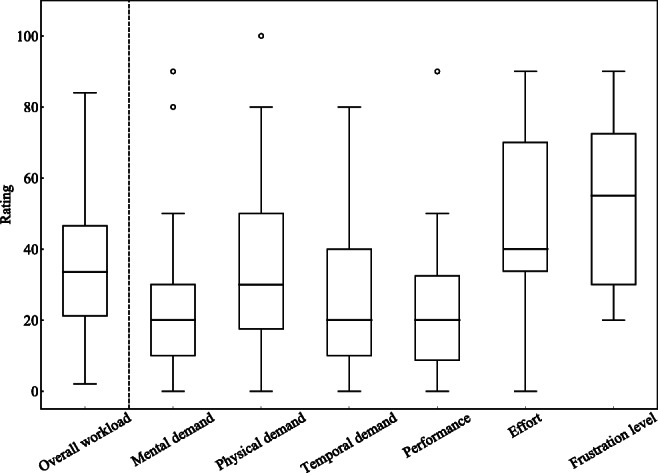
Box plots of NASA-TLX ratings of subscales and overall workloads for all participants

Figure 6 presents results of the subjective workload scores from NASA-TLX. The median overall workload score (33.50) was lower than the scores for medical tasks (50.60; 9.00–77.35) and computer activities (54.00; 7.46–78.00) reported in the literature [19].

The user rating of MR-DVS by the Likert scale is shown in Table 1. Positive feedback was obtained for the dose understandability, hand manipulation, and voice interaction. However, evaluation items for hardware performance (field of view and postural discomfort) did not elicit positive feedback.

## Discussion

More than other real-time dose estimation systems for IR, MR-DVS provides an immersive experience and a detailed depiction of the dose distribution. Although several visualization systems can be used for dose distributions [1–3], MR-DVS can visualize the dose distribution in 3D in front of the user’s eyes. This visualization mode will help procedures proceed smoothly, because the surgeon need not look at a distant monitor to know the dose (Table 2).

MR-DVS is designed to use the patient’s own CT images, but some patients undergo IR without having CT images in clinical practice. A new method for appropriate dose calculation even in that situation is necessary, e.g., an approach that calculates the dose on a numerical human model appearing natural when visualizing them with MR.

MR-DVS can accommodate calculations for any IR room. In this study, air dose distributions were calculated based on our institution’s IR room with Innova 4100-IQ. If the room geometry is built to match the other facilities, then the dose corresponding to that geometry would be calculated and displayed.

Dose visualization using MR can also be used for education. An important benefit of MR is that people can experience an immersive experience while maintaining a view of reality. It can be combined with other clinical training, such as catheterization. However, because the immersive feeling of the current MR system is inferior to VR, the VR education system might be useful when particularly addressing radiation dose education.

Limitations exist for this study. First, we tested only one fluoroscopy system in this study. Nevertheless, the limitation is not crucially important. MR-DVS requires no dedicated connection with a particular fluoroscopy system because the necessary information is retrieved from the number recognition system from a console. Moreover, it does not supported aperture shaping of the x-ray beam. A small change of the aperture shape was not captured well by a web camera. By capturing the fluoroscopy system displays to a computer directly (e.g., using a capture board), aperture shaping can be captured sufficiently.

A second limitation is that MR-DVS currently does not support dynamic deformation of geometries such as radiation shields, respiratory displacement, and body motion. We have not implemented a method to reflect respiratory displacement for skin dose estimation. Furthermore, for air dose estimation, the shields’ location varies widely depending on procedures. Only fixed-point shields can be incorporated into the geometry [5], but it is not practical to previously calculate dose distributions a priori under the conceivable shield locations.

However, postoperatively, it would be possible to calculate the dose considering a shield placed arbitrarily. If the user’s location transferred from the HoloLens to the server was stored, then the situation could be recreated and analyzed during an operation, although, it would be necessary to record the shield’s position in some way. Placing a human numerical model at the stored user’s position in post calculation would also estimate the eye lens dose and other organ doses, such as the thyroid.

A third limitation is that this study was an experimental demonstration of the concept and feasibility of MR-DVS. The clinical use of MR-DVS will require simplifying procedures and developing an integrated dose managing method.

Based on qualitative assessments, MR-DVS enables the user to understand doses quickly. Nevertheless, items related to the usability of HoloLens did not elicit positive feedback. This adverse reaction is attributable to hardware performance bottlenecks that can be improved with the next version of hardware.

The perceived overall workload can be regarded as low. Almost all participants expressed satisfaction. According to the results of qualitative assessment, the workload of the system was small and the understandability of the doses was high. For those reasons, MR technology would be useful for dose visualization for IR.

Positive feedback for dose understandability was not obtained from some participants. Their evaluation will change if they get used to seeing virtual 3D objects with further training. Moreover, MR holographic device performance has been improved. The field of view and the postural discomfort, which did not elicit positive feedback in this study, are expected to be improved in the future. Based on those expected improvements, MR-DVS is expected to become more useful.

## Conclusion

MR dose visualization is expected to improve exposure dose management for patients and health professionals by depicting invisible radiation exposure in real space.

## Supplementary Information

ESM 1 (MP4 79.2 MB)

## Data Availability

Not applicable.
